# Effect of Increasing Assistance From a Powered Prosthesis on Weight-Bearing Symmetry, Effort, and Speed During Stand-Up in Individuals With Above-Knee Amputation

**DOI:** 10.1109/TNSRE.2022.3214806

**Published:** 2023-01-30

**Authors:** Grace R. Hunt, Sarah Hood, Lukas Gabert, Tommaso Lenzi

**Affiliations:** Department of Mechanical Engineering and the Utah Robotics Center, The University of Utah, Salt Lake City, UT 84112 USA; Department of Mechanical Engineering and the Utah Robotics Center, The University of Utah, Salt Lake City, UT 84112 USA; Department of Mechanical Engineering and the Utah Robotics Center, The University of Utah, Salt Lake City, UT 84112 USA; Rocky Mountain Center for Occupational and Environmental Health, Salt Lake City, UT 84111 USA.; Department of Mechanical Engineering and the Utah Robotics Center, The University of Utah, Salt Lake City, UT 84112 USA, Rocky Mountain Center for Occupational and Environmental Health, Salt Lake City, UT 84111 USA.

**Keywords:** Artificial limbs, prosthetic limbs, rehabilitation robotics, sit-to-stand, stand-up, wearable robotics

## Abstract

After above-knee amputation, the missing biological knee and ankle are commonly replaced with a passive prosthesis, which cannot provide net-positive energy to assist the user. During activities such as sit-to-stand, above-knee amputees must compensate for this lack of power using their upper body, intact limb, and residual limb, resulting in slower, less symmetric, and higher effort movements. Previous studies have shown that powered prostheses can improve symmetry and speed by providing positive assistive power. However, we still lack a systematic investigation of the effect of powered prosthesis assistance. Without this knowledge, researchers and clinicians have no framework for tuning powered prostheses to optimally assist users. Here we show that varying the assistive knee torque significantly affected weight-bearing symmetry, effort, and speed during the stand-up movement in eight above-knee amputees. Specifically, we observed improvements in the index of asymmetry of the vertical ground reaction force at the point approximating maximum vertical center of mass acceleration, the integral of the intact vastus medialis activation measured using electromyography, and the stand-up duration compared to the passive prosthesis. We saw significant improvements in all three metrics when subjects used the powered prosthesis compared to the passive prosthesis. We saw improvements in all three metrics with increasing assistive torque levels commanded by the powered prosthesis. We also observed increased weight-bearing asymmetry at the end of movement, and increased kinematic asymmetry with increasing assistance from the powered prosthesis. These results show that powered prostheses can improve functional mobility, potentially increasing quality of life for millions of people living with above-knee amputations.

## I. Introduction

ABOVE-KNEE amputation impacts millions of people worldwide, significantly reducing their movement ability and quality of life [1]. After amputation, the missing biological knee and ankle are replaced by prosthetic knee and ankle joints attached to the user’s residual limb via a socket [2]. These prosthetic joints are typically passive, meaning they cannot generate net positive energy or actively provide assistance during ambulation. The lack of power from the prosthesis contributes to individuals with amputation being generally slower, less stable, and less symmetric than nonamputees. Secondary conditions include osteoarthritis, back pain, and reduced mobility, contributing to reduced quality of life and a high incidence of depression [1], [3], [4], [5]. Improved prosthesis technology is needed to improve the quality of life of individuals with above-knee amputations.

Standing up is a fundamental activity of daily living. Healthy individuals stand up 60 times per day on average [6]. Moreover, standing up is considered the most mechanically demanding functional task routinely undertaken during daily activities [7]. This task is even more difficult for individuals with above-knee amputations [8], [9]; only one knee can generate power. Therefore, amputees must compensate with their intact leg, residual limb, and upper body. In addition to being difficult, standing up is essential for independent living. Standing up is a prerequisite to walking. Without the ability to stand up, one cannot perform essential activities of daily living, such as getting out of bed or using the toilet independently. Difficulty standing up is a major factor limiting the activity of above-knee amputees [10]. Improving the ability of amputees to stand up could improve their functional mobility and quality of life.

Powered prostheses have the potential to make standing up easier for individuals with above-knee amputations [11]. Embedded actuation systems enable powered prostheses to provide net positive energy during ambulation, replacing the biomechanical function of the missing biological limb. Previous studies which used a commercially available powered prosthesis, the Ossur Powered Knee [8], [12], [13], suggested that powered knees in combination with passive ankles do not significantly improve weight-bearing symmetry—an important indicator of balance. In contrast, a study with a research powered knee and powered ankle combined significantly improved weight-bearing symmetry, although some subjects needed to use armrests while standing up, decreasing the reliability of the results [14]. A recent case series with a powered knee and powered ankle has shown improved weight-bearing symmetry and muscle effort during standing-up using a proportional electromyographic (EMG) controller, with the subject directly controlling the amount of assistance at each point in time [15]. However, we still lack essential knowledge about how to tune powered prostheses to best assist users.

Powered prostheses are typically controlled to provide a level of assistance that closely matches the torque produced by healthy legs. Although this approach seems intuitive, it is not necessarily the best. The torque from a powered prosthesis is not transferred to the trunk through the bones and tendons of a healthy limb. Instead, prosthesis torques pass through the socket and the soft tissues of the residual limb [16]. From there, the torque produced by the prosthesis is transferred to the trunk through the user’s residual (prosthesis-side) hip joint, which is typically weakened and less mobile than healthy hips [16]. Because the prosthesis, socket, and residual limb are structurally different from healthy limbs, controlling a powered prosthesis to provide a level of torque that is higher or lower than that of healthy legs may lead to better outcomes. Only three research groups have reported the amount of assistive torque produced by a powered prosthesis during stand-up [14], [15], [17]. No study has systematically investigated multiple levels of torque.

In this study, we tested eight levels of assistance from a powered knee-ankle prosthesis during stand-up. We chose to only change the knee torque while keeping the ankle control consistent because the knee produces higher torques during able-bodied stand-up [18]. We hypothesized that increased levels of knee assistance would improve weight-bearing symmetry, reduce effort, and increase speed during standing up compared to using a passive prosthesis. To test this hypothesis, we asked eight above-knee amputees to stand up with their prescribed passive prostheses and with a powered knee and ankle prosthesis tuned to provide eight different levels of assistance at the knee joint. We used the index of asymmetry of the vertical ground reaction force as the primary outcome measure of weight-bearing symmetry, the integral of the intact vastus medialis EMG as the primary outcome measure of effort, and the time to stand up as the primary outcome measure of speed.

## II. Methods

### A. Participants

We recruited eight individuals with above-knee amputations for this study. Inclusion criteria were unilateral above-knee amputation, daily use of prescribed prosthesis, and ability to stand from a chair without using their hands for assistance. Exclusion criteria included any musculoskeletal, cardiovascular, neurological, or other impairments that would prevent a subject from completing the study activities. More details on the subjects can be found in Table I. The study protocol was approved by the University of Utah’s Institutional Review Board (Protocol 00103197, approved 6/16/2021). Before the experiment began, subjects provided written informed consent and written permission to publish photographs and videos of the experiments. A certified prosthetist was present during all experiments.

### B. Instrumentation

The powered prosthesis used in this study was the Utah Bionic Leg, a battery-operated, lightweight robotic prosthesis with dedicated knee and ankle/foot modules capable of providing high levels of torque. The knee torque was controlled as a function of knee position, using a look-up-table which prescribed an amount of torque at each knee angle based on able-bodied biomechanics [18]. The look-up-table prescribed low levels of torque at the beginning and end of the movement. The peak torque occurred at approximately 85% of the subject’s sitting knee angle after [18]. A virtual damping torque was added to the torque from the look-up-table. To prevent the knee from extending before the user weighted the prosthesis, the knee torque was limited until the ground reaction force (GRF) exceeded 150 N. The ankle was controlled using impedance control, and its equilibrium position was changed linearly from dorsiflexion during sitting to neutral during standing based on the prosthesis knee position. The relationship between the knee and ankle positions was set so that the foot was flat at the start of the movement. Virtual stiffness and damping added flexibility about the desired ankle equilibrium angle. The peak look-up-table knee torque was the independent variable in this study. All knee and ankle coefficients were kept constant across subjects and trials. This controller allows the user to stand up from any height of chair, because the starting knee position is not constrained. For more information, see Supplementary Materials section B.

A 12-camera Vicon motion capture system (Vicon Motion Systems, Centennial, CO, USA) collected 3D motion data at 200 Hz. Two AMTI OR6-7 force plates (AMT, Watertown, MA) recorded GRFs under each foot at 1000 Hz. We placed retroreflective markers (14 mm diameter, Vicon Motion Systems, Centennial, CO, USA) on bony prominences of the study participants, following a modified plug-in-gait marker set to track 15 segments, following [19]. We placed markers for the prosthesis ankle joint axis proximal to flexible ankle components of the passive ankles. We captured a static trial with each prosthesis while the subject stood in the recommended static calibration pose (Vicon Nexus 2.1, Vicon Motion Systems, Centennial, CO). We captured a functional range of motion trial with each prosthesis in order to calibrate functional joint centers [20], [21].

We recorded electromyography (EMG) signals from the vastus medialis of the intact leg. We located the vastus medialis following SENIAM placement procedures [22], shaved the skin over the vastus medialis, and wiped the skin with alcohol. We attached a Delsys Tringo Avanti EMG sensor to the prepared skin using an adhesive. The EMG system was time-synchronized and recorded by Vicon Nexus.

### C. Experimental Protocol

**Fig. 1.**
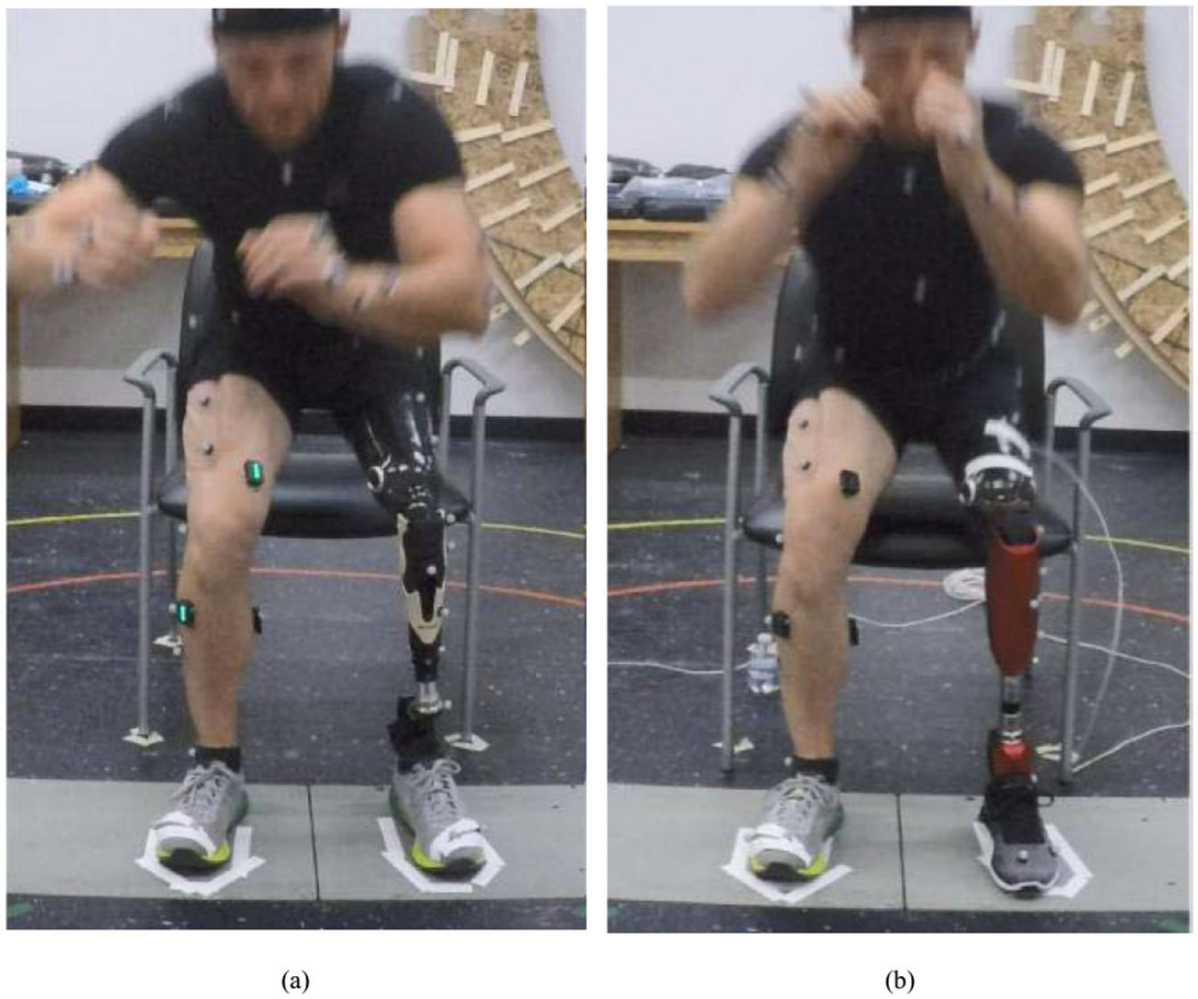
Experimental setup. The subject stands up with feet equally spaced on two force plates, without using their hands. Reflective markers track the movement of the subject’s body segments. (a) The subject stands up with their passive prosthesis. (b) The subject stands up with the powered prosthesis.

First, each subject stood up 8-10 times with their prescribed passive prosthesis, which will be referred to as the “passive trial”. During this passive trial, we asked subjects to sit with both feet equally spaced on two force plates in a comfortable position for stand-up. We outlined their feet with tape. The same foot position was used for the remainder of the study, including powered trials. Subjects were asked to stand up without using their hands for assistance (Fig. 1 (a)).

Next, a certified prosthetist fit and aligned the powered prosthesis to the subject. The subject performed between 8 and 20 practice stand-up movements with different levels of torque. During practice, each subject was instructed to put weight on the prosthesis as needed to benefit from the assistance (see Supplementary Materials section B). When the subject felt comfortable, we started collecting “powered trials”. During each powered trial, we changed the desired peak torque to a different level. Specifically, we used 0.2, 0.4, 0.6, 0.8, 1.0, 1.2, 1.4, and 1.6 Nm/kg. Peak torques were selected to provide multiple levels above and below the able-bodied stand-up peak torque of 0.8 Nm/kg [18]. The order of appearance of the powered assistance levels was randomized for each subject. During each trial, subjects were instructed to perform 8-10 repetitions with their feet equally spaced on two force plates and without using their hands (Fig. 1 (b)).

### D. Data Processing

Vicon Nexus was used to record, synchronize, and post-process marker trajectories and GRFs. Vicon Nexus was used to calculate the functional axes of the knees using symmetrical axis of rotation estimation (SARA) [20], and the functional hip joint centers using symmetrical center of rotation estimation (SCORE) [21]. Data were imported into Visual3D (C-Motion, Germantown, USA) and low pass, fourth order, bidirectional Butterworth filters were applied to the marker trajectories and GRFs, at 6 Hz and 15 Hz, respectively. These cutoff frequencies were based on residual analysis [23]. Visual3D was used to model the body segments and calculate joint angles for the ankles, knees, and hips. The model of the prosthesis shank segment was modified following [24], [25]. We imported the synchronized joint data, EMG, and recordings from the Utah Bionic Leg into MATLAB (Mathworks, Natick, MA). We bandpass filtered the raw EMG at 20 and 450 Hz, rectified, and then filtered again using a low pass, fourth order, bidirectional Butterworth filter at 3Hz. We normalized all EMG envelopes for each subject by the peak of the subject’s passive trial EMG envelope. We calculated secondary joint variables (velocities, accelerations, etc). Finally, we filtered all signals using a low pass, fourth order, bidirectional Butterworth filter at 3Hz.

We identified the start and stop of the stand-up movement, 0% and 100% of stand-up completion, respectively, by thresholding the knee position and velocity. Thresholds were adjusted to accommodate variability in starting and ending knee positions and knee velocities. We detected the start of stand-up when the knee velocity was more negative than a threshold that varied from −10 to −25 deg/s and the knee position was above a threshold that varied from 70 to 94 degrees. We detected the end of stand-up when the knee velocity became more positive than the same velocity threshold and the knee position was below a threshold that varied from 0 to 20 degrees. We segmented each stand-up movement based on the prosthesis knee and the intact knee separately. We used the intact-side knee segmentation for analysis of the EMG from the intact vastus medialis muscle and the intact-side stand-up duration. We performed all other analyses using the prosthesis-side segmentation. We interpolated all variables to the same length for visualization purposes, between −35 and 135% of the movement completion. We calculated all time-dependent metrics, including duration and EMG integral, using non-normalized time measured in seconds.

We calculated the index of asymmetry (IOA) of the vertical GRF, a measure of weight-bearing symmetry, using the following equation after [8], [14], [26]:

(1) $$I\;O\;A=\frac{Prosthesis\;GRF-Intact\;GRF}{Prosthesis\;GRF+Intact\;GRF}*100$$

The index of asymmetry can also be calculated by subtracting the prosthesis side from the intact side, which reverses the sign. We performed this calculation at each point in time, and set the index of asymmetry to zero before subjects stood up, when the IOA is meaningless because there is not sufficient weight on the force plates. For statistical comparisons of index of asymmetry between trials, we extracted the index of asymmetry at the peak of the sum of the right and left GRFs, which approximates the peak vertical acceleration of the center of mass [8], [12], [14].

We calculated the integral of the vastus medialis (VM) EMG as a measure of muscle effort. We calculated this integral between the time points marking −35% and 135% of the intact-side stand-up movement completion, because the EMG contractions started before the intact knee began to move and ended after the intact knee stopped moving. We normalized all of each subject’s EMG integrals by the integral from the passive trial. Therefore, the integral from the passive trial is equal to 1 for all subjects, and the integrals from the powered trials represent an increase (>1) or decrease (<1) in EMG integral, compared to standing up with the passive prosthesis.

We calculated the prosthesis-side stand-up duration as the time between the beginning and end of the prosthesis knee segmentation (0% and 100% prosthesis-side stand-up completion, respectively). We calculated the intact-side stand-up duration as the time between the beginning and end of the intact knee segmentation (0% and 100% intact-side stand-up completion, respectively).

We assessed kinematic asymmetry by normalizing the joint angles of both intact and prosthesis knee joints and both intact-side and prosthesis-side hip joints between 0% and 100% of prosthesis-side stand-up completion. Then we subtracted the prosthesis-side normalized joint angle from the intact-side normalized joint angle at each point in time [27], [28]. Due to prosthesis malfunction, one subject’s asymmetry was an outlier during the 0.2 Nm/kg trial, and all torque trials for this subject were removed from the kinematic asymmetry plot, to allow for easier visualization of the observed trends.

### E. Statistics

To create the figures presented in this paper, we averaged the last 5 stand-up repetitions of each torque level for each subject, resulting in a single mean signal for each variable during each torque level for each subject. Within each torque level, therefore, we had one signal per subject. We calculated means and standard errors at each torque level, across all subjects. In all figures, we show the between-subject means and standard errors. The standard error indicates inter-participant variability.

For all bar plots, statistical comparisons of our three main outcome measures, and all other metrics reported in the text such as peak-to-peak values, we calculated the metric during each individual stand-up movement, and then averaged the metric for the last 5 stand-up repetitions of each torque level, for each subject. Therefore, within each torque level, we had one datapoint per subject. Finally, within each torque level, we calculated the mean and standard error across subjects. The bar height in each bar plot is the between-subject mean, and the error bars represent standard errors. Metrics are reported in the text as (mean ± standard error).

For each of our three primary outcome measures, we tested whether any of the torque levels were significantly different from any other torque levels using a repeated-measures ANOVA with Greenhouse-Geisser corrections for sphericity. The ANOVA tests showed that all three primary outcome measures had at least one torque level that was significantly different (Table II). Therefore, we performed post-hoc pairwise two-tailed t-tests to compare the passive prosthesis trial and the highest assistance trial (1.6 Nm/kg) to each other torque level, for all three primary outcome measures. The significance level *α* was set at 0.05, and p-values were corrected for multiple comparisons using the Bonferroni-Holm method. We chose to only test for differences from passive and from 1.6 Nm/kg, rather than performing all pairwise comparisons, in order to preserve the power of our statistics while still testing for differences between passive and powered, and for differences between levels of powered assistance. We fit exponential curves to the means of the powered assistance levels using nonlinear least squares regression.

## III. Results

### A. Weight-Bearing Symmetry Improved With Assistance

**Fig. 2.**
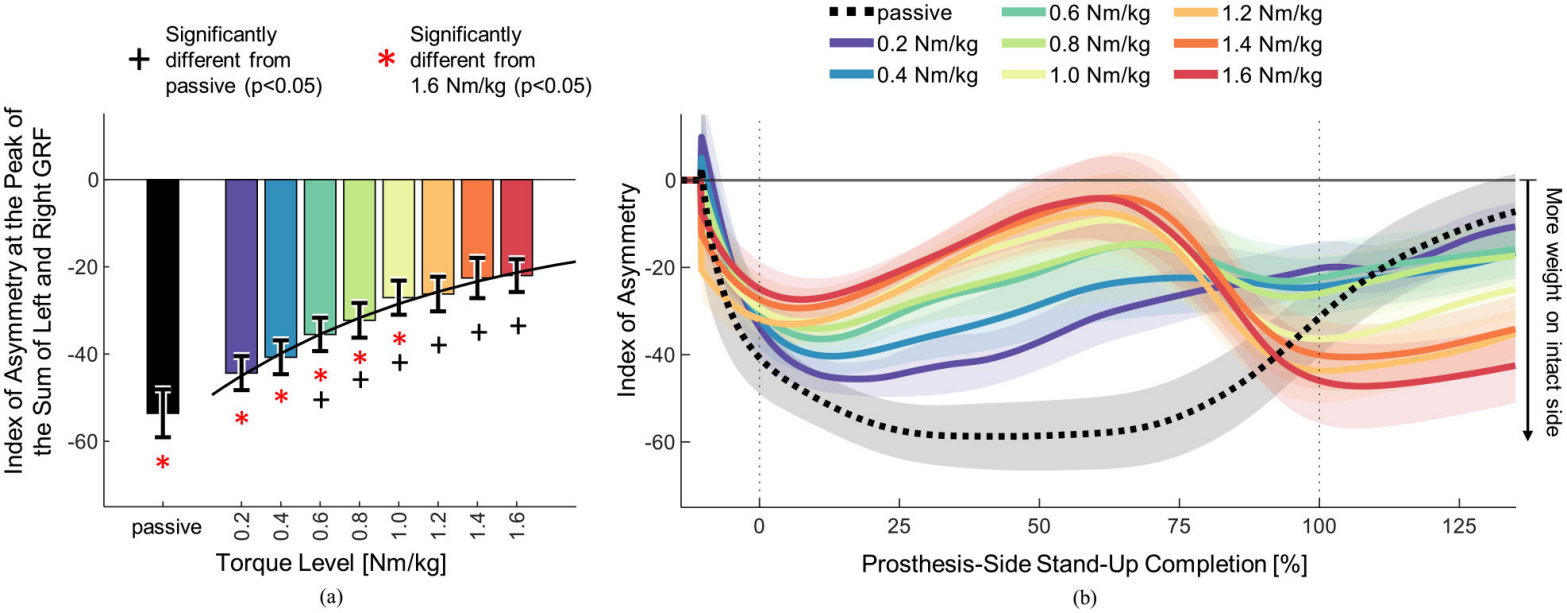
Index of asymmetry of the vertical ground reaction force (GRF), calculated using (1). Negative index of asymmetry indicates more weight on the intact side, and positive values indicate more weight on the prosthesis side. (a) Index of asymmetry at the peak of the sum of right and left GRFs. Bar heights indicate means, error bars indicate standard errors (N = 8 subjects). There was a significant effect of torque level (Table II). Significant differences from the passive trial are indicated with black plus signs. Significant differences from the 1.6 Nm/kg trial are indicated with red asterisks (Table II). Black curve is an exponential regression on the means of the powered torque levels, with the equation *IOA(TL)* = −41.81 *e*^−0.7714*TL*^−9.135. R^2^_adj_ = 0.9861. (b) Index of asymmetry as a function of prosthesis-side stand-up completion, including time before the prosthesis knee movement starts (<0%) and after the prosthesis knee movement ends (>100%). Lines indicate means, shaded regions indicate standard errors (N = 8 subjects).

Weight-bearing symmetry, measured using the index of asymmetry of the vertical GRF at the point approximating peak vertical acceleration of the center of mass, improved significantly when comparing passive to powered, and with increasing levels of powered assistance (paired t-tests, p<0.05) (Table II). The passive trial had the worst (most negative) index of asymmetry (−53.58 ± 5.49), whereas the highest level of assistance (1.6 Nm/kg) had the best index of asymmetry (−22.01 ± 3.76), a 59% improvement. The index of asymmetry for the powered prosthesis trials also improved with increasing levels of assistance (Fig. 2 (a)). The index of asymmetry for the passive trial was significantly worse (less symmetric) than the six highest levels of assistance (Table II). The index of asymmetry for the highest level of assistance was significantly better (less negative) than the asymmetry for passive, 0.2, 0.4, 0.6, 0.8, and 1.0 Nm/kg trials (Table II). Thus, the powered prosthesis significantly improved weight-bearing symmetry compared to passive, and the improvements were greater with increasing assistance.

The index of asymmetry fluctuated substantially during the stand-up movement (Fig. 2 (b)). There were different behaviors between passive and powered trials, as well as between levels of powered assistance. When the movement started, the index of asymmetry was negative during all trials, indicating more weight on subjects’ intact legs (Fig. 2 (b)). During the passive trial, the index of asymmetry continued to decrease and stayed negative for most of the stand-up movement, indicating that subjects placed a majority of their weight on their intact leg during most of the movement (Fig. 2 (b). In contrast, when using the powered prosthesis with the higher levels of assistance, the index of asymmetry increased sharply at the start of the movement and peaked at approximately 70% of movement completion, and then decreased again as the movement completed (Fig. 2. (b)). Notably, with the highest levels of assistance, subjects achieved positive values of index of asymmetry, indicating more weight on the prosthesis side. Thus, higher levels of assistance from the powered prosthesis enabled the subjects to distribute their weight more evenly on both legs during the stand-up movement.

### B. Muscle Effort Decreased With Assistance

**Fig. 3.**
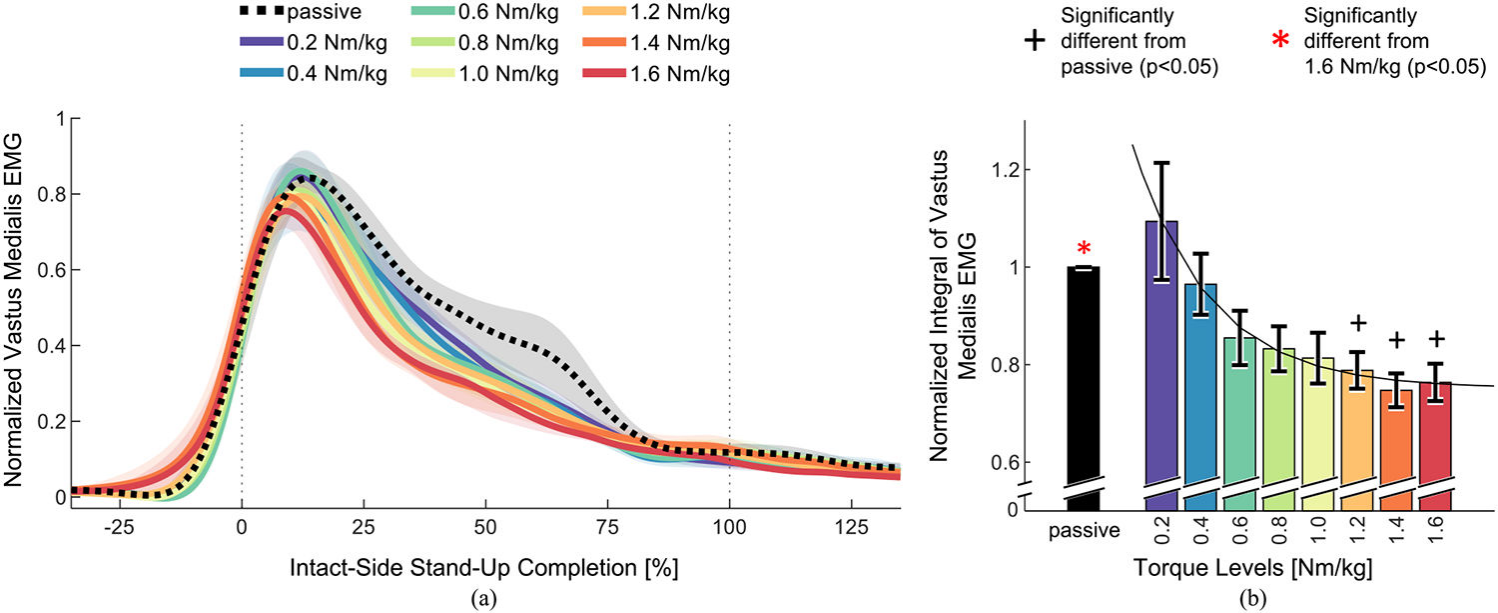
Intact vastus medialis muscle EMG. (a) Normalized vastus medialis EMG as a function of intact-side stand-up completion, including time before the intact knee movement starts (<0%) and after the intact knee movement ends (>100%). Lines indicate means, shading indicates standard errors (N=8 subjects). (b) Vastus medialis EMG integral calculated between −35% and 135% of intact-side stand-up completion, calculated using non-normalized time. Each subject’s EMG integrals were normalized to their passive prosthesis trial. The y-axis is cut between 0 and 0.6 to provide a more detailed view of the region of interest. Bar heights indicate means, error bars indicate standard errors (N=8 subjects). There was a significant effect of torque level (Table II). Significant differences from the passive trial are indicated with black plus signs. Significant differences from the 1.6 Nm/kg trial are indicated with red asterisks (Table II). Black curve is an exponential regression on the means of the powered torque levels, with the equation *VMintegral(TL)* = 0.565*e*^−2.53*TL*^ + 0.751. R^2^_adj_ = 0.9806.

Muscle effort, measured using the intact vastus medialis EMG, significantly decreased when comparing passive to powered, and with increasing levels of powered assistance (paired t-tests, p<0.05) (Table II). The EMG profile was similarly shaped between torque levels (Fig. 3 (a)). However, there were visible differences between 10% and 75% of movement completion, between passive and powered trials as well as between different levels of assistance. The EMG integral with the three highest levels of assistance was significantly lower than with the passive prosthesis (Table II, Fig. 3 (b)). At the highest level of assistance, the EMG integral was significantly lower than with the passive prosthesis (0.76 ± 0.04 versus 1 ± 0), a 23.6% decrease (Table II, Fig. 3 (b)). Therefore, high levels of assistance from the powered prosthesis significantly decreased muscle effort during stand-up compared to passive.

### C. Stand-Up Time Decreased With Assistance

**Fig. 4.**
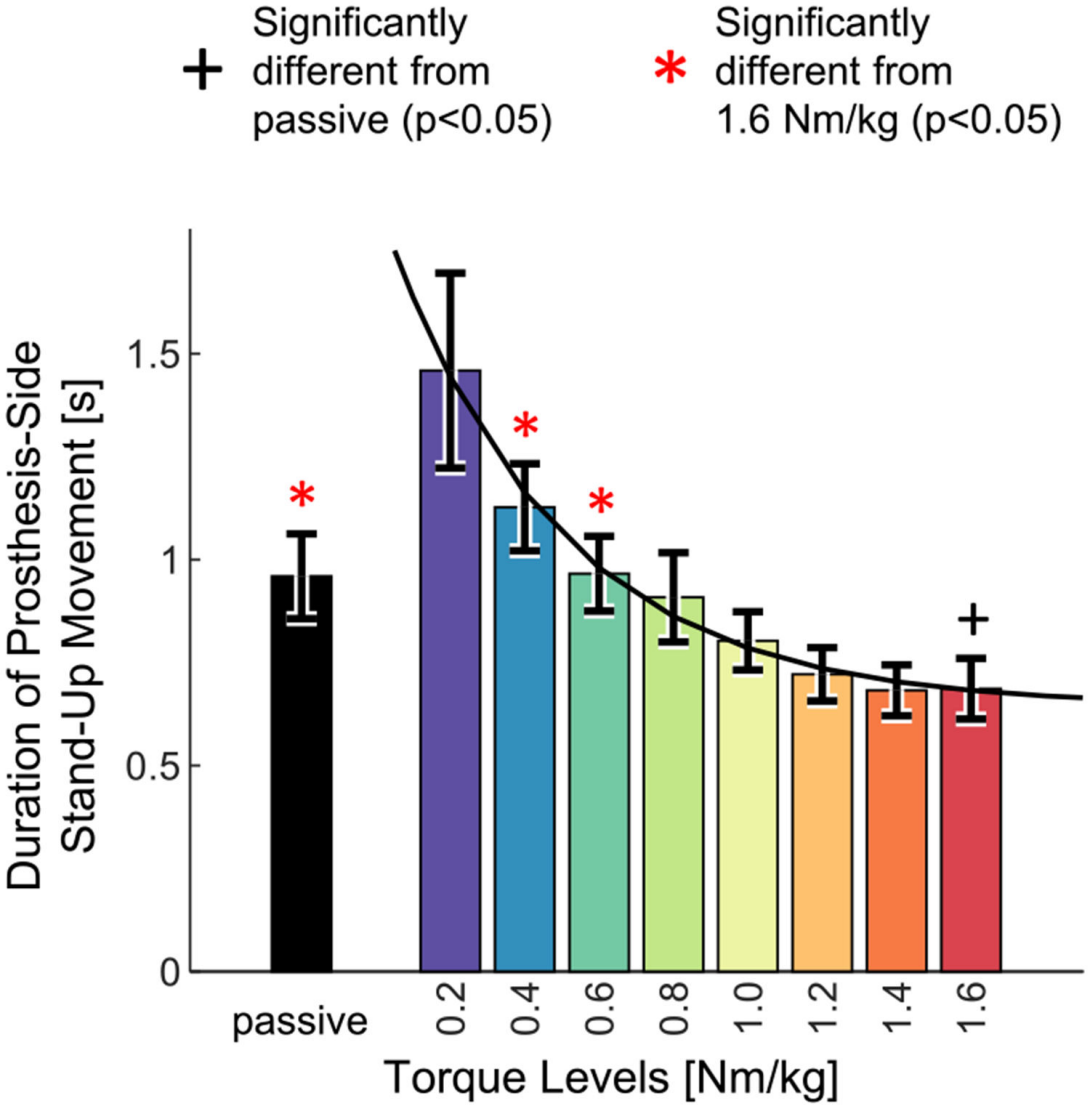
Duration of the prosthesis-side stand-up, measured from 0% to 100% of prosthesis-side stand-up completion. Bar heights indicate means, error bars indicate standard errors (N=8 subjects). There was a significant effect of torque level (Table II). Significant differences from the passive trial are indicated with black plus signs. Significant differences from the 1.6 Nm/kg trial are indicated with red asterisks (Table II). Black curve indicates an exponential regression on the means of the powered torque levels, with the equation *Duration(TL)* = −1.23*e*^−2.167 *TL*^ + 0.644. R^2^_adj_ = 0.9865.

Stand-up time, measured using the prosthesis-side stand-up movement duration, decreased significantly when comparing passive to powered, and with increasing levels of powered assistance (paired t-tests, p<0.05) (Table II, Fig. 4). The lowest level of powered assistance (0.2 Nm/kg) resulted in the longest stand-up movement, 1.46 ± 0.24 s. The passive trial’s duration was 0.96 ± 0.10 s, nearly equal to the duration for 0.6 Nm/kg (0.97 ± 0.09 s) (Fig. 4). The fastest stand-up movement was observed with the highest level of assistance (1.6 Nm/kg) (0.69 ± 0.07 s), 28.1% faster than with the passive prosthesis. Speed increased with increasing levels of assistance, and the durations for passive, 0.4, and 0.6 Nm/kg levels were statistically different from the duration for the highest assistance level (Table II). Thus, the powered prosthesis enabled the subjects to stand up faster than the passive prosthesis, and speed increased with increasing assistance.

### D. Secondary Analyses

The index of asymmetry, the EMG integral, and the stand-up duration all exhibited exponential-like behavior as the level of powered assistance increased. Specifically, all three outcome variables were more sensitive to changes in torque level at lower levels of assistance, and less sensitive at higher levels of assistance. We captured this behavior by fitting exponential curves (2)(3)(4) resulting in $R_{\text{adj}}^{2}$ values of 0.986, 0.981, and 0.987 for the index of asymmetry, EMG integral, and stand-up duration, respectively. The fitted equations were:

(2) $$I\;O\;A\;(TL)=-41.81e^{-0.7714TL}-9.136$$

(3) $$VMintegral\;(TL)=0.565e^{-2.53TL}+0.751$$

(4) $$Duration\;(TL)=1.23e^{-2.167TL}+0.644$$

where TL is the torque level in Nm/kg.

**Fig. 5.**
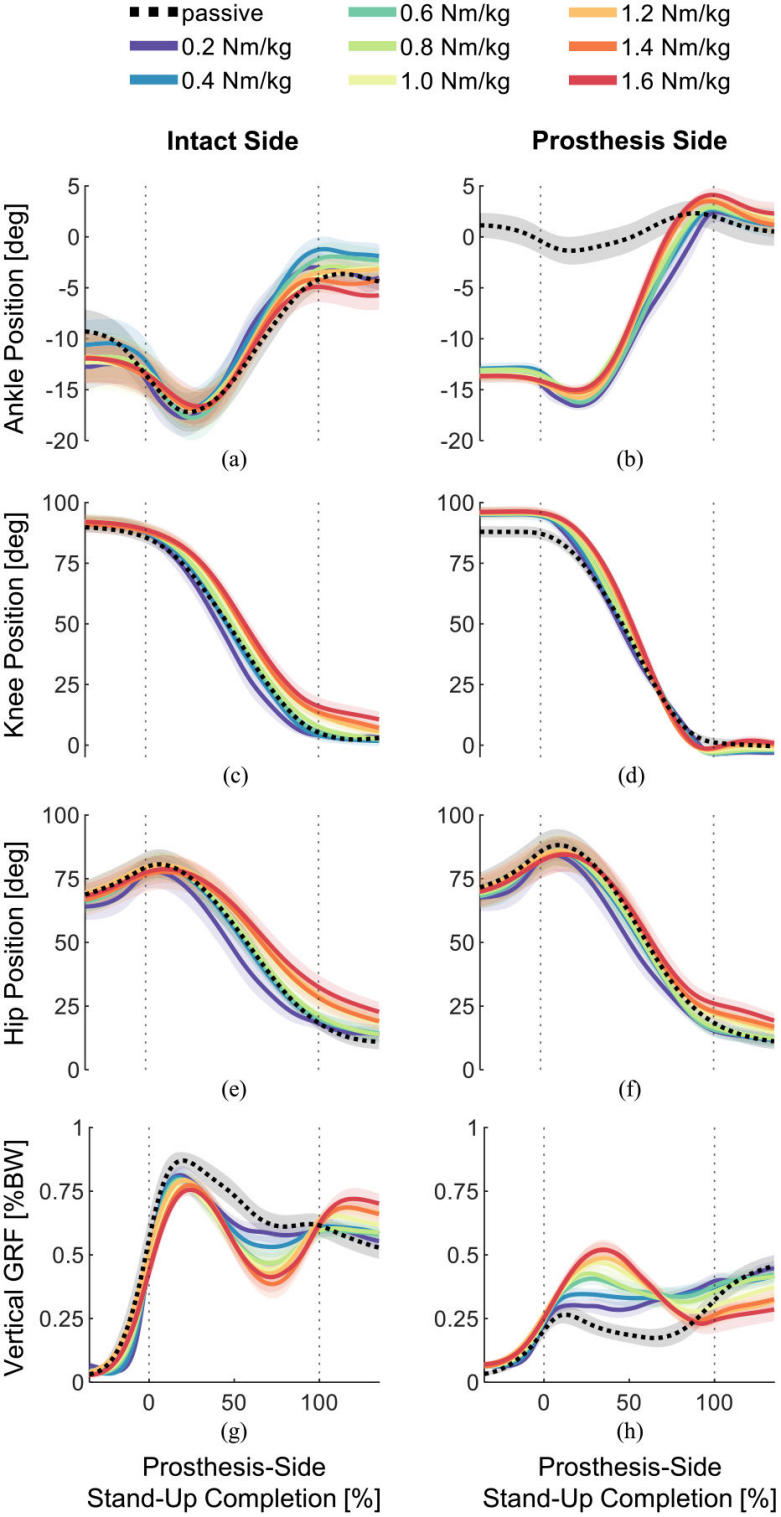
Joint angles and ground reaction forces (GRFs) from intact leg (left column) and prosthesis-side leg (right column). Lines indicate means, and shading indicates standard errors (N=8 subjects). (a) Intact ankle position. (b) Prosthesis ankle position. (c) Intact knee position. (d) Prosthesis knee position. (e) Intact-side hip position. (f) Prosthesis-side hip position. (g) Intact-side vertical GRF normalized to body weight. (h) Prosthesis-side vertical GRF normalized to body weight.

Kinematics and GRFs for intact and prosthesis-sides are shown in Fig. 5. Overall, kinematics appeared similar across torque levels, with some exceptions. The passive prosthesis ankle position differed from the powered prosthesis ankle positions (Fig. 5(b)) because all subjects’ passive ankles were stiff, spring-like, flexible feet without rotating joints. The intact knee, intact-side hip, and prosthesis-side hip showed trends of being further flexed (lagging behind the prosthesis knee) at 100% of prosthesis-side movement completion, and this lag increased with increasing assistive torque (Fig. 5 (c), (e), (f)). The passive trial had the highest intact-side GRF peak (Fig. 5 (g)), and the passive GRF slowly decreased through the movement, while the prosthesis-side GRF slowly increased (Fig. 5 (h)). Comparatively, the powered trials showed decreasing intact-side GRF peaks and increasing prosthesis-side GRF peaks, as well as dynamic “bouncing” with increasing assistance (Fig. 5 (g), (h)).

**Fig. 6.**
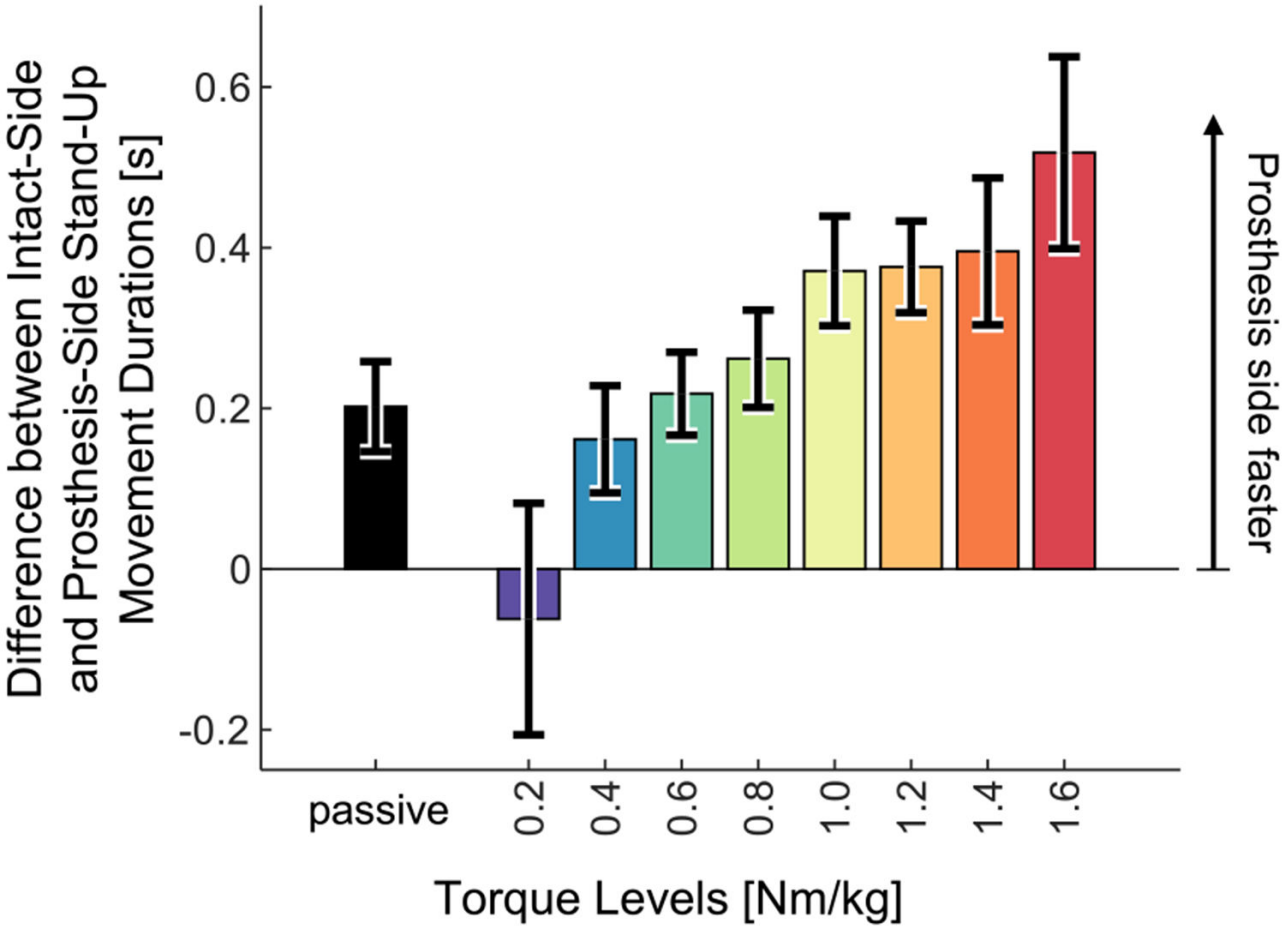
Differences between the intact-side stand-up duration and the prosthesis-side stand-up duration. Positive values indicate that the prosthesis knee performed the stand-up movement in less time than the intact knee.

The intact knee took longer to complete the trial than the prosthesis knee during most trials, and this difference increased with increasing powered assistance. The differences between the prosthesis knee stand-up durations and intact knee stand-up durations are shown in (Fig. 6). Positive values indicate that the prosthesis knee performed the movement in less time than the intact-side knee. The prosthesis finished in less time than the intact leg for all torque levels except 0.2 Nm/kg (Fig. 6). The difference increased with increasing assistance level. For the highest assistance, the prosthesis performed the movement 0.52 seconds faster than the intact leg on average (0.69 ± 0.07 s for 1.6 Nm/kg, versus 1.20 ± 0.16 s for passive).

**Fig. 7.**
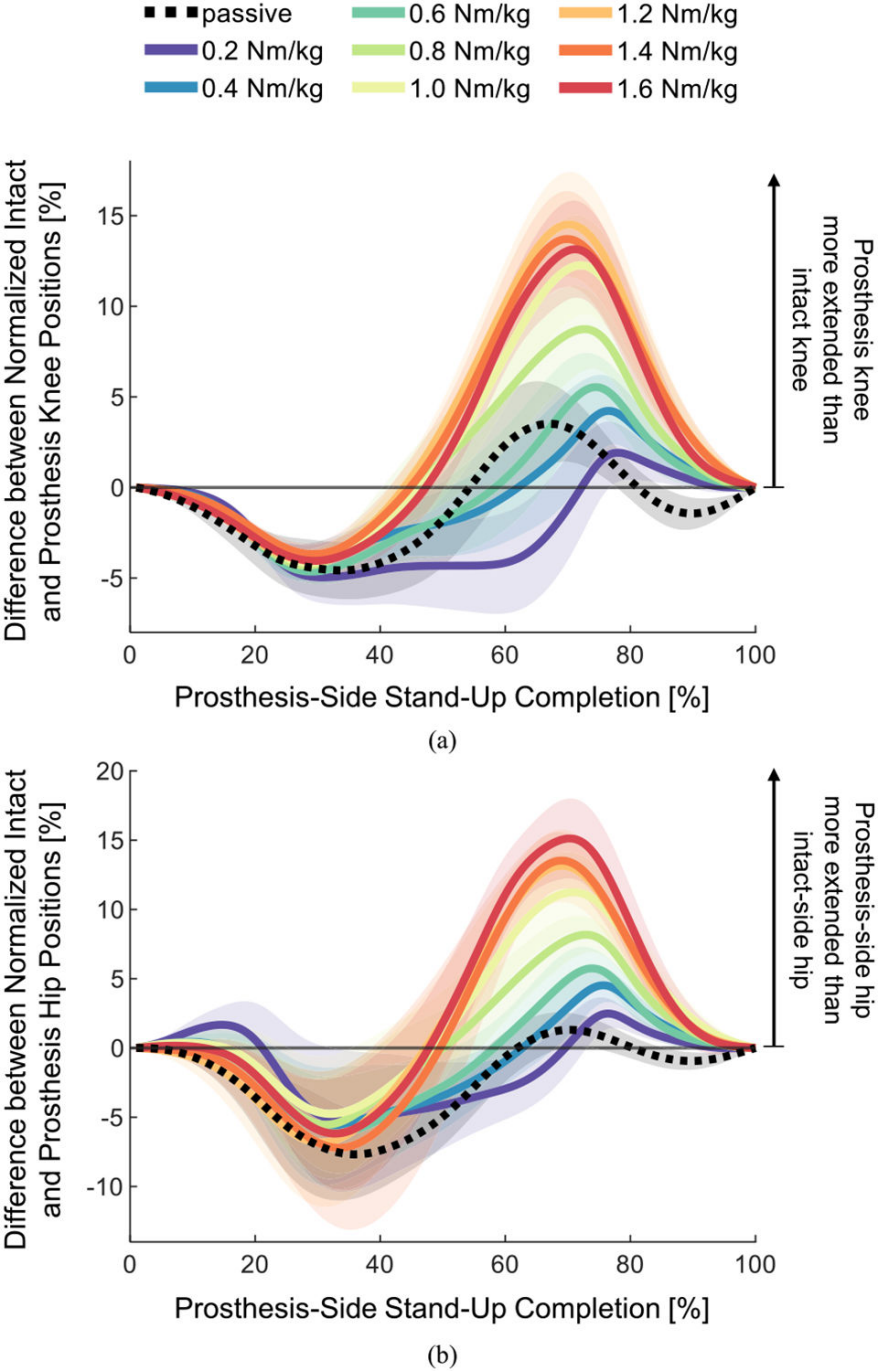
Knee and hip asymmetry measures. Intact and prosthesis-side knee and hip angles were normalized, and asymmetry was calculated as a straight difference between normalized intact and normalized prosthesis positions. Lines indicate means, shading indicates standard errors (N=7 subjects). Positive values indicate that the prosthesis-side joint is more extended (further into the stand-up movement) than the intact-side joint (a) Kinematic asymmetry of the knee joints. (b) Kinematic asymmetry of the hip joints.

The knee and hip joint kinematic asymmetry measures (Fig. 7) showed that the intact-side joints “led” (negative values) at the beginning of the movement, after which the prosthesis-side joints overtook the intact-side joints and “led” (positive values) during the second part of the movement (Fig. 7 (a), (b)). For lower levels of torque, the kinematics stayed more symmetric (closer to zero) throughout the movement, whereas higher levels of torque resulted in large joint asymmetries later in the movement (Fig. 7 (a), (b)). The range of asymmetry during each trial was quantified using peak-to-peak values (maximum – minimum). The peak-to-peak value of the knee joint asymmetry was highest during the 1.6 Nm/kg trial (10.42 ± 0.96 %), and lowest during the 0.6 Nm/kg trial (7.87 ± 1.05 %). The peak-to-peak knee asymmetry during the passive trial was (9.28 ± 0.88 %), lower than the asymmetry during the 0.8, 1.2, 1.4, and 1.6 Nm/kg trials. The peak-to-peak hip joint asymmetry was the highest during the 1.4 Nm/kg trial (15.40 ± 3.98 %), lowest during the passive trial (8.18 ± 1.33 %), and second-lowest during the 0.4 Nm/kg trial (8.78 ± 2.02 %).

## IV. Discussion

Powered prostheses have the potential to improve sit-to-stand in individuals with above-knee amputations. However, the lack of systematic investigations of assistance on standing-up performance is preventing the field from maximizing the clinical benefit of powered prostheses. In this study, we show that increasing the knee extension torque from a powered prosthesis has a significant effect on weight-bearing symmetry and speed, and a visible but non-significant effect on effort. Specifically, our study showed that increasing the level of assistance provided by a powered prosthesis lead to significantly better weight-bearing symmetry (Fig. 2 (a)), reduced muscle effort (Fig. 3 (b)), and significantly shorter standing-up time (Fig. 4) in eight individuals with above-knee amputations. We also found that the highest level of powered assistance, 1.6 Nm/kg, improved all three outcome measures significantly compared to the passive prosthesis. Specifically, weight-bearing symmetry improved by 59%, muscle effort decreased by 23.6%, and stand-up duration was 28.1% shorter with the powered prosthesis’s highest assistance compared to the passive prosthesis. Weight-bearing symmetry reached positive values for some subjects, meaning they put more weight on their prosthesis-side than on their intact-side (Fig. 2 (b)). The observed improvements are substantial and clinically meaningful. Muscle fatigue and weight-bearing asymmetry have both been linked to increased fall risk [29], [30], and time to stand up is commonly used in clinical and rehabilitation settings to assess functional lower limb strength, balance, and fall risk [31], [32]. Our results show that the powered prosthesis outperformed passive prostheses during the standing up movement, and that increasing levels of assistance improved clinical outcome measures.

Although the index of asymmetry improved with higher assistance during a majority of the stand-up, this was not true at the end of the movement. After about 80% of the stand-up completion, when the movement was nearly completed, higher levels of assistance resulted in worse weight-bearing symmetry (Fig. 2). The worse weight-bearing symmetry continued after the movement was completed, when the prosthetic knee joint had reached full extension and the assistive torque was zero. However, when the knee reached the end stop, the joint speed was not zero, and increased with increasing levels of assistance. We speculate that subjects instinctively lifted weight from the prosthesis-side when the prosthesis reached the end-stop at higher speeds. We expect that this behavior could be eliminated if the stand-up controller was updated to provide more damping at the end of the movement so that the prosthetic knee slows before it reaches the end stop. Further experiments are necessary to test this hypothesis.

Although all three primary outcome measures improved as the level of assistance from the powered prosthesis increased, all outcome variables exhibited “diminishing returns”. In other terms, we observed smaller improvements as the assistance level increased, which was captured by exponential curves fit to each outcome measure. We hypothesize that these diminishing returns occur because of difficulty transferring and controlling higher levels of torque through the soft-tissue interface between the residual limb and prosthetic socket using the limited strength of the prosthesis-side hip muscles. We expect that subjects increased their residual hip torque, resulting in higher activations of the residual hip muscles, although we did not measure them in this study. We believe that although the exponential fits seem to explain the observed trends, they should not be used to predict user performance outside of the tested conditions. In fact, increasing the assistance beyond the levels tested in this study could degrade performance. Researchers and clinicians should weigh tradeoffs between small improvements in performance against the “cost” of providing higher torques, which requires larger batteries and motors. The diminished returns to increased levels of assistance is a critical factor that should be considered in the tuning of powered prostheses.

We also observed that kinematic asymmetry increased with increasing assistance. Kinematic asymmetry was minimized at lower levels of assistance (0.6 Nm/kg for the knee and passive for the hip). In contrast, weight-bearing symmetry was maximized at the highest level of assistance (1.6 Nm/kg). Thus, the level of assistance that maximized weight-bearing symmetry was different from the level of assistance that maximized kinematic symmetry. Although kinematic symmetry may be a valuable outcome for some activities like walking, in our opinion, weight-bearing symmetry is a much better predictor of performance during sit-to-stand. During sit-to-stand, both feet share the body weight during the entire movement to lift the person’s mass vertically. In a perfectly balanced stand-up movement, the kinematics and kinetics would both be symmetric. However, in above-knee amputees, prosthesis alignment and soft-tissue deformation introduce inherent kinematic asymmetries. During prosthetic alignment, the location of the knee joint is adjusted so that the prosthetic knee is locked in extension and does not buckle during standing [16]. Therefore, the standing angles of the intact knee and prosthetic knee are different, causing kinematic asymmetry during the standing up movement. Moreover, the prosthesis is connected to the residual limb through soft tissues which deform proportionally to the torque applied by the prosthesis to the socket [16]. Thus, when a powered knee joint generates extension torque to lift the subject, the soft tissues will deform and the knee and hip kinematics will change.

Generalization of these results to other powered prostheses depends on both the maximum speed/torque capabilities of the device and the specific assistive control strategy. The controller used in this study allows for the peak assistive torque to be set directly. In contrast, other controllers may define the prosthesis knee torque using a combination of stiffness and fixed or variable equilibrium angles [14], [17]. To match the result of this study, the control parameters could be tuned so that the resulting assistive torque is similar to the levels used in this study. This process may require subject-specific tuning but would allow researchers to generalize our results to other powered devices and related controllers.

Although the ankle behavior was not systematically changed, we believe that pairing powered knee prostheses with powered ankles is key to improving sit-to-stand performance, which is reflected in the fact that no studies of powered knees with passive ankles have shown significant improvements in weight-bearing symmetry [8], [12], [13]. In intact limbs, the ankle joint plays an important role during stand-up movements [33] by dorsiflexing to allow for a more posterior foot position and more flexed knees before the stand-up movement starts, which makes the movement easier [34]. Unfortunately, a posterior foot position is very difficult to achieve with passive prosthetic ankles. Most prosthetic ankle/foot prostheses are stiff, flat springs, which are difficult to dorsiflex [35]. Therefore, above-knee amputees must decide between starting stand-up with their prosthetic foot anterior and flat on the ground, or posterior and balanced on the “toe” of the prosthesis [10], both of which may cause them to over-exert their intact joints to compensate for the sub-optimal starting position. In contrast, powered ankles can actively dorsiflex before the stand-up movement begins and allow the user to select a natural starting position. This functional difference may be the underlying reason why the only significant improvements in weight-bearing symmetry and muscle effort during stand-up with powered prostheses have been shown in studies with powered knees combined with powered ankles [14], [15]. Future studies should investigate the role of the ankle by controlling the ankle torque based on able-bodied ankle torque profiles, including testing increased ankle torque with and without corresponding increases in knee torque.

### A. Limitations

One limitation of the controller used in this study is that the peak prosthesis knee torque was normalized based only on the subject’s weight. The optimal assistance may also vary based on other subject-specific factors, such as height, residual limb length, and mobility level. Our previous study using volitional EMG control showed that when above-knee amputee subjects were in direct control of their assistive torque, the peak torques were significantly affected by factors such as chair height, speed of the movement, and additional weight carried by the subject [15]. The controller used in this study does not currently adapt to these factors, but could be adapted in future work. Adapting prosthesis movements based on subject factors, environmental factors, and activity modification could improve the real-world viability of powered prostheses.

A second limitation of the controller used in this this study is that the behavior of the powered ankle was kept constant. Our control strategy allows for synchronized movement of the powered knee and ankle joints while providing users with the freedom to control the speed of the stand-up movement. However, it does not allow for direct control of the assistance provided by the ankle joint during standing up, which may improve performance during standing up. Future studies should directly investigate the function of ankle joint assistance during stand-up movements in order to investigate the need for a powered ankle joint and determine optimal prosthesis ankle joint functions and tuning.

In this study, subjects did not receive extensive training with the powered prosthesis. None of the subjects had previous experience using the powered prosthesis with the proposed controller for sit-to-stand, although five subjects had experience using the current or previous version of the Utah Bionic Leg for other research activities. After the powered prosthesis was donned and aligned, subjects performed 8-20 practice stand-ups at different assistance levels. In contrast, all subjects in this study used their prescribed passive prosthesis daily, and therefore had much more experience with their passive device. Training and practice are important factors in prosthetic proficiency, because it takes time to build confidence and trust the prosthesis. Previous studies have shown that performance with prosthetics improves with practice [36]. One case study observed that outcomes continued to improve weekly for at least three weeks after a transfemoral amputee was fitted with a new knee [37]. A study of 10 transtibial amputees fitted with new ankles showed improved outcomes at multiple time points, with significant improvements observed between 60 and 90 day check-ins [38]. Therefore, we expect that with more practice, subjects’ confidence would improve and allow them to put more weight on the prosthesis and lift less weight with their intact leg, improving weight-bearing symmetry and muscle effort even more than we observed during this study.

Finally, we did not measure subjective preference. Many of the subjects reported that standing up with the powered prosthesis was easier. In general, subjects seemed to prefer an intermediate level of assistance, and some subjects reported that the highest levels of assistance felt too fast, especially when higher levels were presented early in the data collection. However, all subjects became increasingly comfortable with higher levels as the trial progressed. Future studies should include a systematic investigation of subjective preference. We believe that the user’s personal preferences are paramount for the success of powered prostheses.

## Supplementary Material

supp1-3214806

## Figures and Tables

**TABLE I:** Subject Information

Sex	Age [years]	Weight [kg]	Height [m]	Age of Amputation [years]	Amputation Side	Reason for Amputation	Previous Experience with Utah Bionic Leg	Passive Knee Prosthesis	Passive Ankle Prosthesis	Socket Suspension
M	29	65.14	1.778	8	R	Trauma	Y	Plie	All Pro	Suction
M	52	102.27	1.905	13	L	Trauma	Y*	C-Leg	Triton	Pin Lock
M	64	88.64	1.880	5	L	Trauma	N	Rheo	Proflex XC	Vacuum
F	26	68.18	1.753	7	R	Trauma	N	Plie	Proflex XC	Suction
M	38	100.45	1.803	10	L	Trauma	N	C-Leg	All Pro	Suction
M	40	90.45	1.905	35	L	Trauma	Y	Plie	Soleus	Suction
F	32	59.09	1.600	12	L	Trauma	Y*	Plie	All Pro	Lanyard
M	31	77.27	1.803	4	L	Infection	Y	Plie	Freedom Hi	Suction

* asterisks = subject has used the previous version of the Utah Bionic leg, but had not used the current version before participating in this study

**TABLE II:** Results of Statistical Tests

Index of Asymmetry of Vertical GRF				
Repeated-measures ANOVA	GG-corrected df Torque Level	GG- corrected df error	F stat	GG- corrected p-value
	2.65	18.55	34.33	1.575e-07*
Pairwise two-tailed t-tests	Torque Level being compared	Bonferroni-Holm corrected p-values		
		vs. Passive		vs. 1.6 Nm/kg
	Passive	-		0.0004668*
	0.2 Nm/kg	0.09847		6.35e-06*
	0.4 Nm/kg	0.09422		0.0004668*
	0.6 Nm/kg	0.02992*		0.005014*
	0.8 Nm/kg	0.005209*		0.002735*
	1.0 Nm/kg	0.003573*		0.02992*
	1.2 Nm/kg	0.002041*		0.05153
	1.4 Nm/kg	0.001071*		0.6641
	1.6 Nm/kg	0.0004668*		-
Integral of Vastus Medialis EMG				
Repeated- measures ANOVA	GG-corrected df Torque Level	GG- corrected df error	F stat	GG- corrected p-value
	1.60	11.19	7.04	0.01366*
Pairwise two-tailed t-tests	Torque Level being compared	Bonferroni-Holm corrected p-values		
		vs. Passive		vs. 1.6 Nm/kg
	Passive	-		0.006389*
	0.2 Nm/kg	1.842		0.3152
	0.4 Nm/kg	1.025		0.1328
	0.6 Nm/kg	0.3152		0.8107
	0.8 Nm/kg	0.1011		0.4911
	1.0 Nm/kg	0.1011		0.8526
	1.2 Nm/kg	0.01021*		1.391
	1.4 Nm/kg	0.002466*		1.842
	1.6Nm/kg	0.006389*		-
Duration of Prosthesis-Side Stand-Up				
Repeated- measures ANOVA	GG-corrected df Torque Level	GG- corrected df error	F stat	GG- corrected p-value
	1.43	10.03	7.61	0.01412*
Pairwise two-tailed t-tests	Torque Level being compared	Bonferroni-Holm corrected p-values		
		vs. Passive		vs. 1.6 Nm/kg
	Passive	-		0.03466*
	0.2 Nm/kg	0.7307		0.1709
	0.4 Nm/kg	0.7766		0.01665*
	0.6 Nm/kg	1.663		0.04091*
	0.8 Nm/kg	1.594		0.06844
	1.0 Nm/kg	0.7766		0.7307
	1.2 Nm/kg	0.1709		1.594
	1.4 Nm/kg	0.06474		1.663
	1.6 Nm/kg	0.03466*		-

N = 8 subjects, p-values marked with asterisks are significant at α = 0.05. df stands for degrees of freedom. All t-tests have 7 degrees of freedom. GG stands for Greenhouse-Geisser corrections for sphericity.
