# Supplementary material for: Effect of Increasing Assistance From a Powered Prosthesis on Weight-Bearing Symmetry, Effort, and Speed During Stand-Up in Individuals With Above-Knee Amputation
Source: IEEE Trans Neural Syst Rehabil Eng. Author manuscript; Available in PMC 2023 Apr 7. (PMC10079568; doi:10.1109/TNSRE.2022.3214806)
Supplement: supp1-3214806 [file NIHMS1869870-supplement-supp1-3214806.pdf]

I. Supplementary material

A. The Utah Bionic Leg

The powered prosthesis used in this study was the Utah Bionic Leg, a self-contained, battery-operated, lightweight robotic leg prosthesis. The Utah Bionic Leg has dedicated knee and ankle/foot modules capable of providing biomechanically accurate torque during ambulation. To achieve high torque in a compact and lightweight device, the powered knee modules use a novel torque-sensitive actuator that combines the benefits of variable transmission [39] with that of series-elastic actuators [40]. The powered ankle/foot modules have two articulated joints, the ankle and the toe, which are powered by a single actuator using a compliant underactuated mechanism. A custom instrumented pyramid adapter is located at the top of the ankle module to estimate the vertical ground reaction force and the torque in the sagittal plane [41]. The knee and ankle/foot modules are mechanically connected with a standard prosthetic pylon, which is cut to size for each user, as in commercially available prostheses. A digital communication line passes through the pylon enabling the knee and ankle modules to synchronize their movements during ambulation. Combined, the powered knee and ankle/foot modules weigh only 3.2 kg including batteries and protective covers.

The Utah Bionic Leg uses a hierarchical control system enabling different ambulation modes. The desired ambulation mode is selected manually by the experimenter through a graphical interface. The high-level controller uses a finite-state machine with a specific number of states and transition rules for each ambulation mode. The middle-level controller uses different control algorithms for each state to generate either a desired joint torque or joint position. The low-level controller translates the desired joint torque or position into current commands for the knee and ankle/foot motors. For both the knee and ankle/foot modules, the position controller is based on a simple proportional-derivative (PD) compensator with additional feed-forward terms for gravity, inertia, and friction compensation. The desired torque is translated into motor current commands using specific algorithms for the knee and ankle modules. This hierarchical control strategy has been shown to enable individuals with unilateral above-knee amputations to walk [42], [43], ascend and descend stairs [44], [45].

B. Stand-Up Controller

At a high-level, prosthesis knee torque was controlled as a function of knee position, and the ankle position was controlled as a function of knee position.

1) Knee Joint Controller

The knee extension torque τ_k (1) was determined by adding torque from a lookup table, τ_{lookup}, which is a function of knee position θ_k, and torque from damping, $\tau_{damping}$, which is a function of knee velocity $\dot{\theta}_k$. This combined torque was then scaled by a factor G which depended on the ground reaction force (GRF) sensed by a load cell in the prosthesis (3).

$$\tau_k = \left[\tau_{lookup}(\theta_k) + \tau_{damping}\left(\dot{\theta}_k\right)\right] * G \qquad (1)$$

τ_{lookup}, the torque from a lookup table, was based on the knee torque and knee position at each point in time during able-bodied stand-up, extracted from [18]. The knee torque and knee position at each point in time was extracted and entered into two look-up tables. The first look-up-table, LUT1, defined the knee torque at knee angles between the sitting knee position θ_{k_sit} and the knee position at peak torque θ_{k_peak}. The second look-up-table, LUT2, defined the knee torque at knee angles between the knee position at peak torque θ_{k_peak} and the knee position at standing, θ_{k_stand} (2). Both look-up-tables were scaled so that the knee peak torque in each LUT was equal to 1. To use these look-up tables, the measured knee angle was used to look up the corresponding knee torque from the LUT, which was then multiplied by the desired peak torque τ_{peak}.

$$\tau_{lookup} = \begin{cases} 0 & \theta_k > \theta_{k\_sit} \\ LUT1(\theta_k) * \tau_{peak} & \theta_{k\_sit} > \theta_k > \theta_{k\_peak} \\ LUT2(\theta_k) * \tau_{peak} & \theta_{k\_peak} > \theta_k > \theta_{k\_stand} \\ 0 & \theta_k < \theta_{k\_stand} \end{cases} \qquad (2)$$

Both LUTs could be scaled using the graphical user interface to specify the knee position at the start of stand-up (θ_{k_sit}, adjusted based on each patients' sitting knee angle), knee position at the end of stand-up (θ_{k_stand}, always set to 5 degrees), and position at which peak torque occurred (θ_{k_peak}, always set to 85% of the subject's sitting knee position).

The knee torque was additionally modulated at the beginning of the movement, based on the weight detected by an on-board ground reaction force (GRF) sensor. A multiplier, G, was applied to the output torque as seen in (1). Before the GRF exceeded a first threshold (50Nm), G equaled 0, and no output torque was applied. When the GRF was between the first threshold and the second threshold, G was increased linearly with the GRF, between 0 and 1, and a corresponding proportion of the desired output torque was be applied. When the GRF was above the second threshold (150 Nm), G equaled 1, and the full desired output torque was be applied (7).

$$G = \begin{cases} 0 & GRF < F_{low} \\ \dfrac{GRF}{F_{high} - F_{low}} & F_{low} < GRF < F_{high} \\ 1 & GRF > F_{high} \end{cases} \qquad (3)$$

Knee torque from damping, $\tau_{damping}$ was calculated as the knee joint velocity $\dot{\theta}_k$ multiplied by the knee virtual damping coefficient B_k (4). B_k was 0.01 Nm/deg/s during a majority of the movement. Between 0 and 5 degrees of knee extension, $B_k$was increased to 0.1 Nm/deg/s in order to slow the knee as it approached the endstop. To avoid a "step", $B_k$was increased linearly from 0.01 to 0.1, starting at 6 degrees, and ending at 5 degrees (5).

$$\tau_{damping} = -B_k * \dot{\theta}_k \qquad (4)$$

$$B_k = \begin{cases} 0.01 & \theta_k > 6 \\ 0.01 + 0.09 * (6 - \theta_k) & 5 < \theta_k < 6 \\ 0.1 & \theta_k < 5 \end{cases} \qquad (5)$$

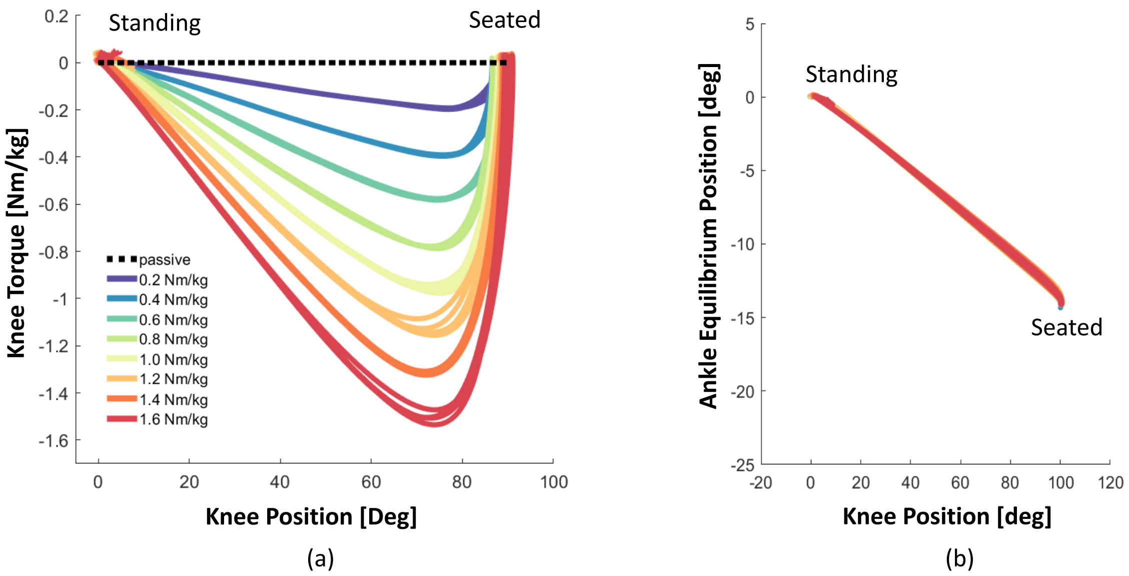

Supplementary Figure 1: Overview of desired output for the powered knee (a) and powered ankle (b), as recorded by the powered prosthesis. (a) The powered knee torque was controlled as a function of the knee position, based on a look-up-table which prescribed a certain amount of torque at each knee position, plus a small contribution of virtual damping torque. The look-up-table peak height could be adjusted to the desired output torque. Representative traces of desired torque for each of the eight levels of torque are shown, as a function of knee position. At zero degrees, when the knee is extended and the subject is standing, the torque is zero. At the maximum knee position, when the knee is flexed and the subject is sitting, the torque is zero. The dotted black line represents the passive condition, where the passive prosthesis injects zero torque during the entire movement. (b) The desired equilibrium position of the ankle was controlled as a function of knee position, in an impedance controller with virtual stiffness and damping. When the subject is standing, the knee position is zero (extended) and the ankle position is zero (neutral). When the subject is seated, the knee position is maximal (flexed) and the ankle position is dorsiflexed (negative). A linear relationship is defined between sitting and standing. Representative traces of all eight powered torque levels are included in this plot, but the ankle behavior was not changed across trials and so the traces overlap.

2) *Ankle Joint Controller*

The prosthesis ankle torque τ_a was controlled using an impedance controller, with an equilibrium position θ_{a_eq} that varied based on the position of the knee θ_k (6).

$$\tau_a = K_a * \left(\theta_a - \theta_{a\_eq}\right) + B_a * \dot{\theta}_a \qquad (6)$$

The virtual stiffness coefficient K_a was set to 2 Nm/deg. The virtual damping coefficient B_a was set to 0.2 Nm/deg/s. θ_a is the measured knee angle, and $\dot{\theta}_a$ is the measured knee velocity.

The ankle equilibrium position θ_{a_eq} was varied based on the position of the knee θ_k, to move from a dorsiflexed ankle position θ_{a_sit} during sitting when the knee was flexed θ_{k_sit}, to a neutral ankle position of zero during standing when the knee was extended θ_{k_stand}:

$$\theta_{a\_eq} = -\frac{\theta_{a\_sit}}{\theta_{k\_stand} - \theta_{k\_sit}} * \left(\theta_k - \theta_{k\_stand}\right) \qquad (7)$$

All damping and stiffness coefficients were determined when the controller was originally designed, and were kept the same for all subjects and all tested conditions in this study.

At the beginning of the study, the subject was asked to move forward on the chair until their shin was angled slightly, with knee approximately over the toe, and θ_{a_sit} was adjusted so that the prosthesis foot was flat on the floor.
